# Biology, Germination Ecology, and Shade Tolerance of Alkaliweed (*Cressa truxillensis*) and Its Response to Common Postemergence Herbicides

**DOI:** 10.3390/plants12142679

**Published:** 2023-07-18

**Authors:** James Schaeffer, Kurt J. Hembree, Anil Shrestha

**Affiliations:** 1 Department of Plant Science, California State University, Fresno, CA 93740, USA; 2 University of California Cooperative Extension, Fresno, CA 93710, USA

**Keywords:** Convolvulaceae, perennial cropping systems, postemergence herbicides, semi-arid environments, weed management

## Abstract

Alkaliweed (*Cressa truxillensis*) is a native perennial plant of the western USA and in California, they are found primarily in saline and alkaline soils. Lately, it has been observed in Central Valley pistachio, olive, and almond orchards as a problematic species. Very little information is available on the effect of environmental factors on germination, shade tolerance, and the response of this species to herbicides. Therefore, studies were conducted to assess the effect of environmental factors (water potential, salinity, and pH) on seed germination, the influence of shade (30% shade 70% shade, and no shade) on aboveground growth, and the response of alkaliweed to common registered post-emergent herbicides. Results showed that the seeds were moderately tolerant to drought but highly adapted to salinity and pH as germination occurred up to an electrical conductivity level of 20 dS m^−1^ and pH range of 5 to 9. Both shade levels reduced aboveground growth and formation of reproductive structures. None of the postemergence herbicides provided adequate control of the plants. Therefore, an integrated management plan needs to be developed for alkaliweed control in Central Valley orchards.

## 1. Introduction

**Figure 1:**
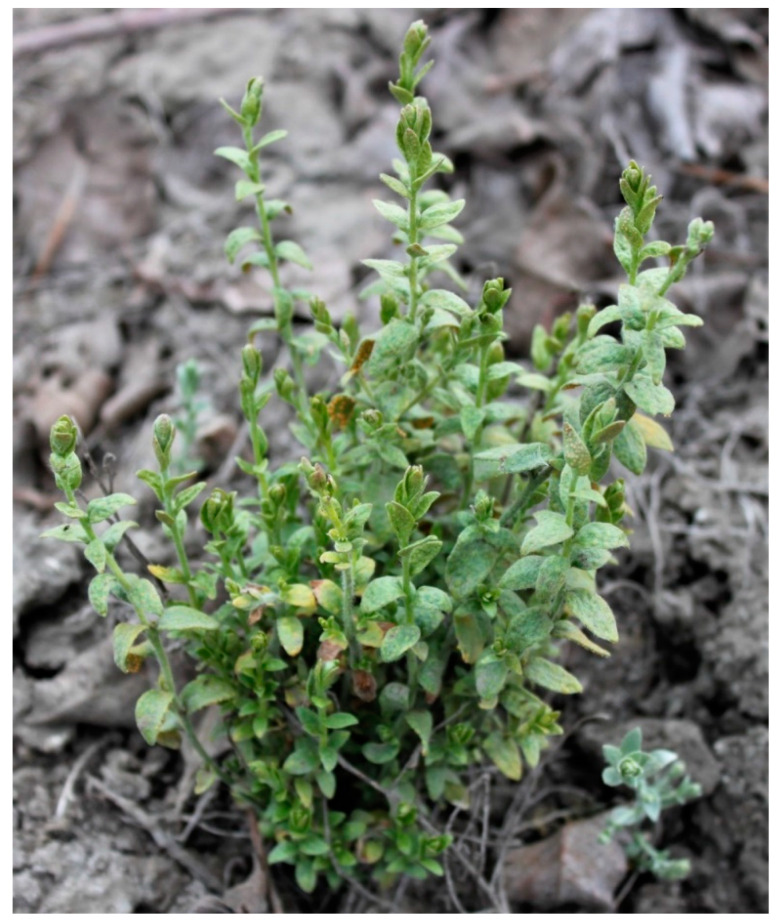
An alkaliweed plant growing in a pistachio orchard in late spring.

Alkaliweed (*Cressa truxillensis*) is a perennial herb (Figure 1) native to the western USA and commonly found in California, USA [1,2]. In California, this species traditionally inhabits wetlands and communities such as yellow pine forests, foothill woodland, chaparral, valley grassland, alkali sink, and wetland riparian [1]. Alkaliweed has primarily been associated with natural areas, field margins, and ditch banks. This species is also reported to be used in revegetation projects in saline and alkaline soils in California [3]. However, in recent years, alkaliweed has been observed to spread by canals and waterways, shifting populations into agricultural areas. Alkaliweed was first reported as a weedy pest to the University of California Cooperative Extension office in 2016 and is now being widely observed in tree nut orchards, agronomic crops, fallow fields, ditch banks, and roadsides. More noticeably, numerous young pistachio (*Pistacia vera* L.) orchards in the southern San Joaquin Valley of California have been completely invaded by this plant, causing a weed monoculture in infested fields. Standard orchard floor management practices, such as between-row cultivation and herbicide application to tree rows, have not resulted in any substantial control of the species (personal communications, Kevin Brooks, Pest Control Advisor). Very little information is available on the biology and ecology of this species and there are no publications on management strategies for this species.

**Figure 2:**
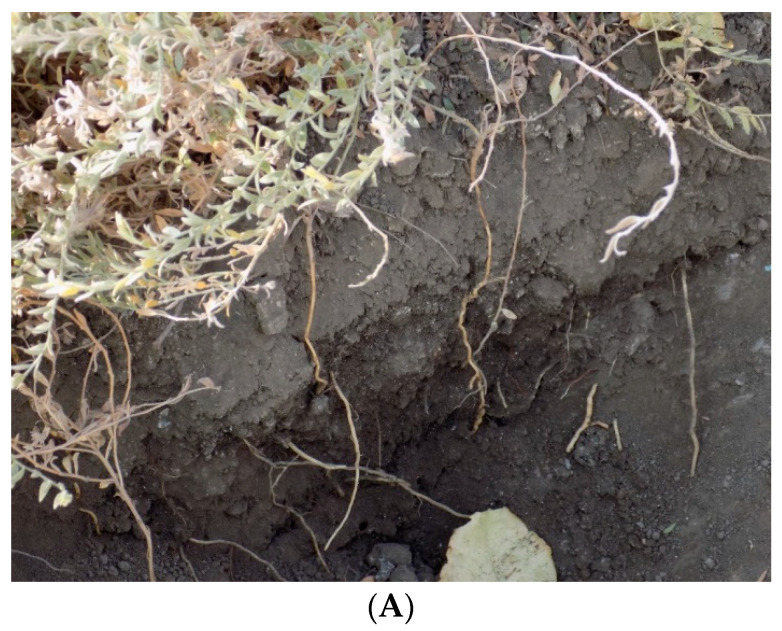

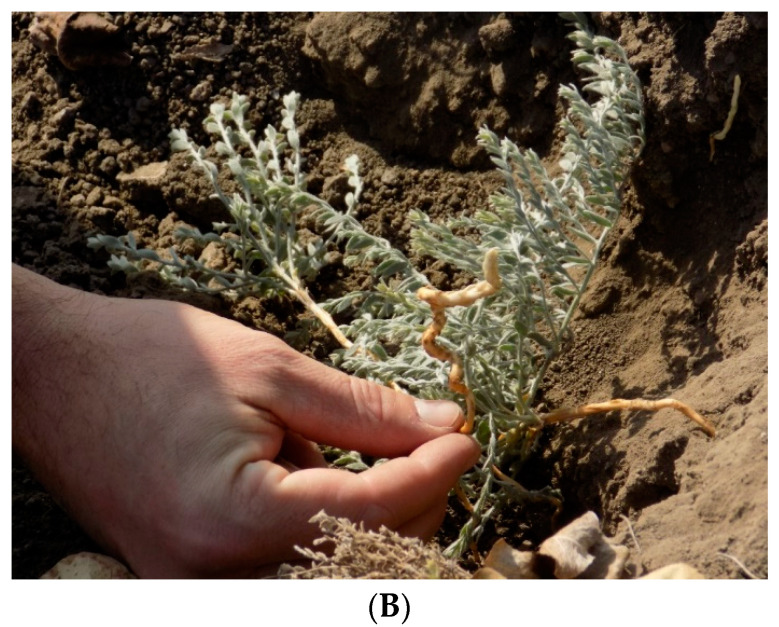
(**A**) Belowground plant parts of alkaliweed, and (**B**) rhizome-like structures of alkaliweed.

Common herbicide treatments used for orchard floor plant management such as glyphosate, glufosinate, salflufenacil, paraquat, 2,4-D, halosulfuron, carfentrazone, rimsulfuron, and oxyfluorfen only appear to suppress treated plants for approximately 30 days before plant regrowth occurs (personal communications, Mr. Kevin Brooks, Pest Control Advisor). Observations at sites with alkaliweed infestations in pistachio orchards in Kings County, CA showed that regrowth of herbicide-treated plants seemed to occur from new stems emerging from extensive underground root and/or shoot systems, as well as from the aboveground parts of the treated plant (Figure 2A,B).

**Figure 3:**
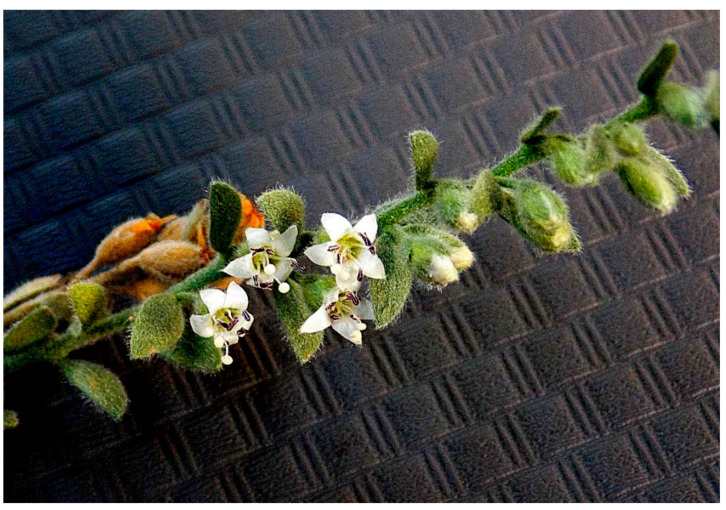
Reproductive structures of alkaliweed.

Similar observations were noted with plants that had been cultivated with mechanical equipment. The need for effective management strategies is paramount as grower concern builds. Anecdotal evidence and observations lead one to suspect that alkaliweed persists due to both its dense aboveground and underground plant parts and rhizome-like structures. While flowers (Figure 3) and seeds are produced, little is known about their contribution to invasion and spread.

Very little is known about the germination ecology of alkaliweed seeds, specifically in response to environmental stresses caused by soil pH, salinity, and moisture. There is one study that looked at seed scarification as a method to improve the germination of this species [3]. The same study also evaluated the effect of salt stress on seed germination. In addition, very little is known about its asexual reproductive ability. The study that focused on environmental parameters required for seed germination did not discuss the asexual reproductive ability of this species [3]. The populations of alkaliweed in Kings County were observed in moderately alkaline and saline soils prone to summer drought. Notably, alkaliweed has been regarded to be shade-intolerant [4]. Observations made over two years (2016–2018) showed that populations of alkaliweed seem to prefer and grow better in full sunlight than in shaded conditions. Plants were seen growing throughout young orchard floors (little tree canopy shading), but in older orchards, alkaliweed was observed mainly in the middle of the rows between trees and along adjacent roadsides and canal banks where sunlight is more available.

Most plant species seem to grow better at high incident photon flux density (PFD) than at low PFD and they are genotypically adapted to open sunny habitats, in general, having the capacity to acclimate and grow in shaded conditions of lower PFDs [5]. This has also been observed to be the case with alkaliweed but has not been confirmed and neither has their morphological response been studied. Therefore, alkaliweed morphology and acclimation to shade need to be explored to help explain their adaptation in orchards with varying shade levels on the floor. Such information may be beneficial in mitigating population spread and in the development of management strategies for this species. Photosynthetic, morphological, and growth responses to the light environment can be useful measurements to determine favorable habitat conditions for the conservation of endangered species [6]; however, such data on alkaliweed may prove to aid in managing populations in agricultural areas and for conservation in natural habitats.

Monteith [7] established the concept of radiation/light use efficiency and explained that it is a function of plant species and can differ between species based on several factors such as crop architecture, etc. Plants seldom grow by themselves and are usually in mixed stands in fields and natural environments. As such, they must compete for growth resources like light to survive and for their fitness in the environment [8]. Therefore, measurements of light environments and adaptations of species may be useful in creating ways to manipulate the desired environment to discourage an increase in population by either suppression, control, or encouraging preventative measures during pre-plant preparations of fields that host favorable conditions. In shade-intolerant plants like *Arabidopsis*, reduction in the red-to-far-red (R:FR) ratio of light has been reported to have numerous effects on plant growth and development. The specific absorption rate (SAR) in *Arabidopsis* was characterized by increased hypocotyl, stem, and petiole elongation, a more erect leaf position, increased apical dominance, and early flowering [9]. Furthermore, Amthor [9] reported that the plants in shaded environments responded differently than those in full sunlight, and morphological differences can be distinguished. Stems are elongated with noticeable increases in internodal length. In addition, leaves are greater in size and plants grow more upright, towards the light, as opposed to their normal bushy herbaceous characteristic. Genetic and photobiological studies performed in *Arabidopsis* have shown that these light sensors mediate numerous adaptive responses (e.g., phototropism and shade avoidance) and developmental transitions (e.g., germination and flowering). Some physiological responses are specifically triggered by a single photoreceptor, but in many cases, multiple light sensors ensure a coordinated response. Alkaliweed is observed as being a shade-intolerant species. If flower and seed production are influenced by shaded conditions in this species, then more options become available in developing management practices to limit its sexual reproduction that contributes to seedbanks. Other aspects to consider, aside from the aboveground growth, are the influences on the belowground growth and partitioning of carbohydrates. The belowground reproductive plant organs have been referred to as ‘bud banks’ [10]. These include structures such as rhizomes, tubers, bulbs, corms, and bulbils. Although the propagation of alkaliweed is primarily by seed [4], field observations have shown sprouting ability by underground plant parts that resemble rhizomes or stems. However, it is not known for certain what the reproductive potential of these structures is in alkaliweed. Therefore, this needs to be studied because limiting both seedbanks and bud banks may be necessary to effectively manage this species. It has been stated that bud banks could serve as demographic storage sites and help species stabilize their population dynamics against various disturbances, enabling them to successfully invade and dominate aboveground plant species [11]. This statement may explain the dominant species situations observed with alkaliweed in the pistachio orchards.

The need to consider belowground resource acquisition is well recognized, even where light requirements are thought to be the primary factors influencing plant communities [12]. Alkaliweed has an extensive, deep underground system of shoots and roots that may be contributing to the production of more plants; however, as mentioned earlier, this is largely unknown, and neither is the effect of shade on these structures known. In an experiment on an Appalachian herb (*Houstonia montana*), it was reported that plants responded markedly to shading, with less total growth and nitrogen accumulation and proportionately more allocation aboveground in the shade compared with full sun. Shading significantly reduced stem number, stem mass, and leaf mass, but increased plant height. Total aboveground and belowground biomasses were reduced by shading. Belowground biomass was reduced proportionately more than aboveground, producing significantly lower root:shoot ratios in the shade compared with the sun treatment [12]. In the pistachio orchards, alkaliweed, under shaded conditions, seemed to show similar responses in the aboveground growth as described in this aforementioned study. The observations in the belowground parts during preliminary explorations of alkaliweed need further study. The spread of alkaliweed by the movement of the belowground parts may be occurring but is not known for certain. Developing data on belowground growth and carbon allocation along with aboveground morphology in response to shading may be an important facet in future management strategies as well as determining potential crop competition. Similarly, it is also important to investigate seed germination in response to environmental stresses to estimate the potential of sexual propagation of this species.

The objectives of this study were to: (i) assess the germination of alkaliweed seeds in response to environmental stresses such as pH, salinity, and moisture, (ii) assess the impact of different levels of shade (full sunlight, 70% full sunlight, and 30% full sunlight) on the aboveground growth and morphology of alkaliweed, and (iii) assess the effect of postemergence herbicides on alkaliweed suppression.

## 2. Materials and Methods

Seed germination experiments were conducted in a controlled-environment growth chamber (Conviron CMP 6010) located at the Jordan Agriculture Research Center (JARC), California State University, Fresno, CA, USA. The temperature was set at 24 °C, relative humidity at 50%, and ambient light in the chamber at a 12 h day/night cycle. These conditions were based on optimum temperatures for several species in the *Convolvulaceae* family.

### 2.1. Preparation of Test Solutions

Solutions of pH 5, 6, 7, 8, and 9 were prepared using sodium hydroxide (NaOH) and hydrochloric acid (HCl) proportions. The solutions were prepared by mixing deionized (DI) water, 1 M HCl, and 1 M NaOH to obtain the desired pH level. The pH level was calibrated with a pH meter (Go Direct^®^ pH Sensor, Vernier, Beaverton, OR, USA).

A range of salinity solutions measured by electrical conductivity (EC) of 0, 2.5, 5, 10, 15, 20, and 25 dS m^−1^ was prepared by using laboratory-grade sodium chloride (NaCl). Solutions of 0 g (g), 1.46 gg, 2.42 g, 5.84 g, 8.77 g, 11.69 g, and 14.61 g of NaCl in 1000 mL of DI water were prepared to create the corresponding concentrations for each EC treatment according to the procedures used by Shrestha et al. [13]. The EC levels were calibrated with an electrical conductivity meter (FieldScout Direct Soil Meter, Spectrum Technology Inc., Aurora, IL, USA).

A series of water potential (ψ) solutions (0, −0.51, −1.88, −2.89, −4.12, and −5.56 MPa) were prepared using polyethylene glycol (PEG 6000). The targeted ψ solutions were prepared by mixing 10 g, 15 g, 20 g, 25 g, 30 g, or 35 g of PEG 6000 each in 50 mL DI water [13,14]. The ψ was calibrated with a thermocouple psychrometer (HR 33-T Dew Point Microvoltmeter connected to a C-52 Sample Chamber, Wescor Inc., Logan, UT, USA).

### 2.2. Germination Tests

Alkaliweed seeds were obtained from a commercial vendor (S&S Seed Inc., Carpenteria, CA, USA). Twenty-five alkaliweed seeds for each individual treatment were manually scarified by using a metal file and placed on seed germination paper (Blue Blotter Circle seed germination paper, Anchor Paper Co., St. Paul, MN, USA) in 100 mm × 15 mm (diameter and depth) Petri dishes. Once seeds are placed in separate Petri dishes, 10 mL of the corresponding treatment solutions (pH, electrical conductivity, water potential) were added into the Petri dish with a pipette. Petri dish lids were sealed with a parafilm (Parafilm M^TM^ Wrapping Film, Fisher Scientific, Houston, TX, USA) and then placed in the growth chamber with the environmental settings as described above. The pH, electrical conductivity, and water potential studies were conducted separately but using similar protocols as discussed below.

The Petri dishes were labeled with treatment and replication numbers before placing them in the chamber. Each treatment was replicated four times and the experiment was repeated. The experiment for each parameter was set up as a completely randomized design. The first round of the experiments was conducted in June/July 2019 and the second in August of 2019. Treatments were monitored in the growth chamber for approximately 14 days, during which the numbers of germinated seeds were counted. A seed was considered to be germinated when a 1 mm or longer radicle was observed. Germination was evaluated every day until seeds ceased to germinate. Usually, there was no germination after 10 days. When a seed germinated, it was counted and removed from the Petri dish using tweezers, and the data were recorded.

### 2.3. Effect of Shade on Growth of Alkaliweed

An alkaliweed-infested roadside (alongside the top of a ditch bank) in Stratford, CA, USA (36°10′29.5″ N, −119°51′10.6″ W) adjacent to a 7-year-old pistachio orchard infested with alkaliweed was used to study the effects of full sun and shade on alkaliweed morphology and growth. The study was conducted on the roadside away from the orchard to avoid any shading effects of the trees. The roadside with very little traffic inside the orchard property was chosen for the study to avoid any dust effects on the plants. Polyvinyl chloride (PVC) pipe frames were constructed and fitted with shade clothes (Gemplers Farm and Home Supply Co., Janesville, WI, USA) representing 30% shade (70% of full sun) and 70% shade (30% of full sun). Frames were 1.8 m long, 0.9 m wide, and 0.33 m tall. A full sunlight treatment with no shade was also included. The experimental shade treatments were set up before alkaliweed plants emerged from the soil. Each treatment was replicated four times in a randomized complete block design. The photosynthetically active radiation (PAR) level inside the tents was taken every week between 11 a.m. and 1 p.m. using a hand-held quantum sensor (LightScout Quantum Sensor, Spectrum Technologies Inc., Aurora, IL, USA). The PAR level was also measured in the full sun treatment. The number of plants and the number of plants with flowers were counted at the onset of flowering. The plants were harvested once five plants within a random 0.36 m^2^ (0.6 by 0.6 m) area frame within each treatment plot had flowered. The plants were clipped to the soil surface, put into paper bags, and transported to the lab at Fresno State. In the lab, the fresh weights of the five plants per treatment were taken, and the number of flowers and internodes on each plant were counted. The internode length on each plant was measured and recorded. The total leaf area on each plant was measured using an LI-COR LI-3000C leaf area meter. The parts of the five plant parts were put together in separate paper bags for each treatment and placed in a forced-air oven set at 60 °C for 72 h and the dry weights were recorded. The experiment was conducted once from April to August 2019. The study could not be repeated due to resource limitations and some logistics of conducting the study in a management-intensive private property.

### 2.4. Effect of Postemergence Herbicides on Alkaliweed

The effect of postemergence herbicides on alkaliweed was tested by spraying actively growing plants on the edge of the road outside a pistachio orchard in Stratford, CA, USA on 11 April 2018. The experiment was not conducted in the actual orchard rows because several herbicides and treatments mentioned below are not labeled for use in pistachio in California. In addition, the study was not repeated due to the same reasons mentioned for the shade study. However, preliminary studies (data not shown) were conducted in 2017 for herbicide treatment selections. Since the plants had more of a semi-prostrate growth habit, the plants were approximately 3 cm tall and 15 cm in diameter at the time of the spray. The herbicide treatments included one-time applications, sequential applications, and tank mixtures of herbicides as shown in Table 1. Adjuvants were added to the herbicide treatments as recommended by each herbicide label and the solutions in all the treatments were buffered to a pH of 5.5 using BioLink^®^ acidifier (Westbridge Agricultural Products, Vista, CA, USA). The herbicides were applied with a two-nozzle (Turbotwinjet 11004) spray boom using a CO_2_ backpack sprayer calibrated to spray 323.6 l ha^−1^ at 4.8 km h^−1^. The spray pressure and spray height were maintained at 30 psi and 45 cm above the plant, respectively. Each treatment plot was 9.1 m long and 1 m wide. The experimental design was a randomized complete block with four replications of each treatment. The alkaliweed plants were visually evaluated at weekly intervals up to 28 days after treatment (DAT) for injury/mortality on a scale of 0 to 100, where 0 was considered completely healthy without necrotic symptoms (no injury) and 100 was considered entirely injured (dead) with no green tissue remaining.

### 2.5. Data Analysis

Data for treatment effects and their interactions with the experimental run in the seed germination studies were analyzed using general linear model procedures in SAS v 9.4. When the ANOVA showed a significance at α = 0.05, the means were separated using Fisher’s Least Significant Difference tests. The germination data failed to meet the assumptions of ANOVA; therefore, they were arcsine square root transformed prior to analysis. The germination data for water potential and EC were further subjected to nonlinear regressions using a three-parameter sigmoidal model (germination= a/(1 + exp(− (x − x0)/b)) by using the drc package in RStudio version 4.2.2. The water potential and the EC levels at which germination was reduced by 50% were also estimated by the drc package. Graphs were prepared in SigmaPlot version 11.0. The data for the two experimental runs in the germination studies were combined and analyzed as there were no interactions (*p* > 0.05) between the experimental runs and treatments.

Data for the shade response and the herbicide treatment study were also analyzed using general linear model procedures in SAS v 9.4 and when the ANOVA showed a significance at α = 0.05, the means were separated using Fisher’s Least Significant Difference tests.

## 3. Results and Discussion

### 3.1. Effect of Water Potential on Seed Germination

**Figure 4:**
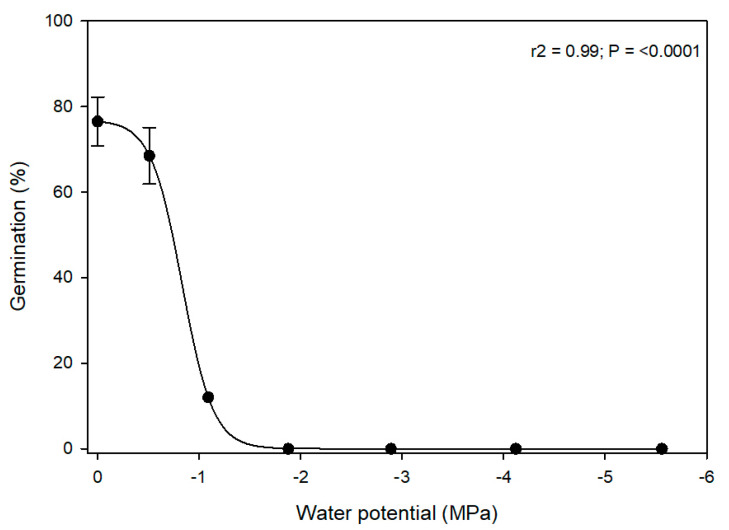
Germination (mean ± SEM) of alkaliweed seeds as a function of polyethylene glycol (PEG)-induced water potential levels. The solid line represents the estimated three-parameter non-linear sigmoidal curve.

Alkaliweed seeds were moderately drought tolerant as germination was 68.5% at −0.51 MPa and 12% of the seeds germinated at a fairly high negative water potential of −1.09 MPa (Figure 4). There was no germination beyond water potential values of −1.09 MPa. The water potential level that reduced seed germination by 50% was estimated by the non-linear regression model as −0.78 MPa.

Although there are no studies that have reported the effect of water potential levels on alkaliweed, there are examples from other species in the *Convolvulaceae* family. For example, it has been reported that the germination of tall morningglory (*Ipomoea purpurea*) was less than 15% at a water potential level of −0.4 MPa [15]. Similarly, a study estimated the water potential that reduced germination by 50% in three-lobe morningglory (*Ipomoea triloba*) as −0.35 MPa [16]. In field bindweed (*Convolvulus arvensis*), the water potential that reduced germination by 50% was estimated as −0.4 [17]. Therefore, it appears that alkaliweed seed is more tolerant to water potential stress than that of other *Convolvulaceae* species during germination, enabling it to germinate in the semi-arid regions of the Central Valley of California.

### 3.2. Effect of Salinity Levels on Seed Germination

**Figure 5:**
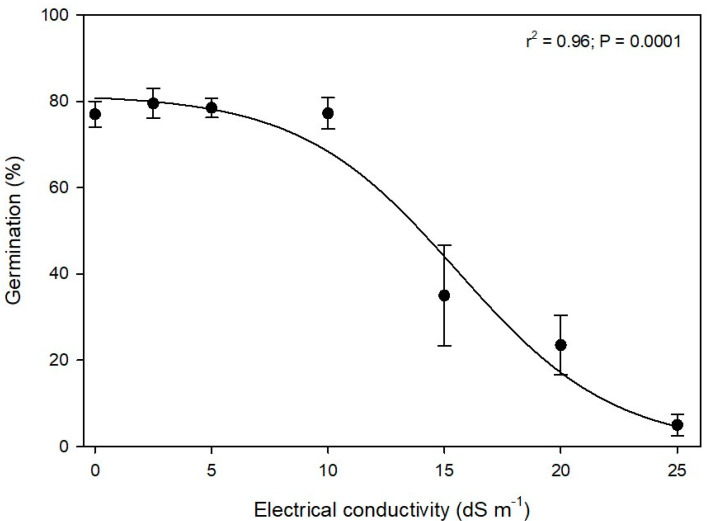
Germination (mean ± SEM) of alkaliweed seeds as a function of sodium chloride (NaCl)-induced salinity concentrations (electrical conductivity). The solid line represents the estimated three-parameter non-linear sigmoidal curve.

Alkaliweed seeds were very tolerant to salinity (sodium chloride) stress at germination as approximately 24% of the seeds germinated at a very high EC level of 20 dS m^−1^, although percent germination was significantly lowered at EC levels higher than 10 dS m^−1^ (Figure 5). The salinity level that reduced seed germination by 50% was estimated by the non-linear regression model as 15.5 MPa.

There is only one study that has reported the effect of salinity levels on alkaliweed and it showed that 300 mM (approximately 30 dS m^−1^) delayed seed germination [3], but in our study, the highest level of salt concentration tested was 25 dS m^−1^ (approximately 250 mM). However, there are examples from other species in the Convolvulaceae family. For example, it was reported that the germination of tall morningglory was less than 15% at a salinity level of 200 mM (approximately 20 dS m^−1^) [15]. Similarly, it has been reported that the germination of three-lobe morningglory was unaffected even at salinity levels as high as 250 mM (approximately 25 dS m^−1^) [16] whereas, in field bindweed, it was determined that a salinity level of 165.3 mM (approximately 165 dS m^−1^) reduced seed germination by 50% [17]. Therefore, it appears that alkaliweed is similar in tolerance to salinity stress as other Convolvulaceae species during germination, enabling it to germinate in the high salinity regions of the west side of the Central Valley of California.

### 3.3. Effect of pH Levels on Seed Germination

**Figure 6:**
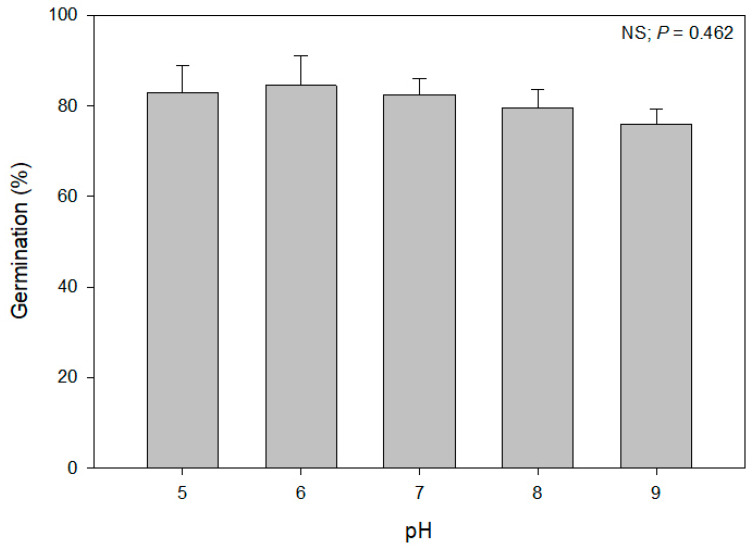
Effect of pH levels on the germination (mean ± SEM) of alkaliweed seeds.

Unlike water potential and salinity, the germination of alkaliweed seeds was not affected by the range of the pH levels tested (Figure 6). Germination was similar at all pH levels (5 to 9) and ranged from 76 to 84%, indicating that this species has a wide range of adaptation to pH levels during germination. Although more than 80% of the seeds germinated at a pH level of 5, it is not known if the plants would grow and reproduce at this pH level because the optimum pH range for growth of this species is reported as 6.8 to 9.2 [4]. Nevertheless, our study showed that this species can possibly germinate in the acidic soils that occur in many areas of the east side of the Central Valley.

While there are no published studies on the germination of alkaliweed seeds in response to environmental factors, there are examples from other species in the *Convolvulaceae* family. For example, it was reported that field bindweed germinated at a pH range of 4 to 9 but the optimum pH was 6 to 8 [17]. Although alkaliweed showed similar results as this study, an optimum pH range could not be determined because the germination percentage was similar at all levels of pH tested. Therefore, it appears that alkaliweed can germinate in the alkaline and acidic soils of the Central Valley of California.

### 3.4. Effect of Shade on Growth of Alkaliweed

**Figure 7:**
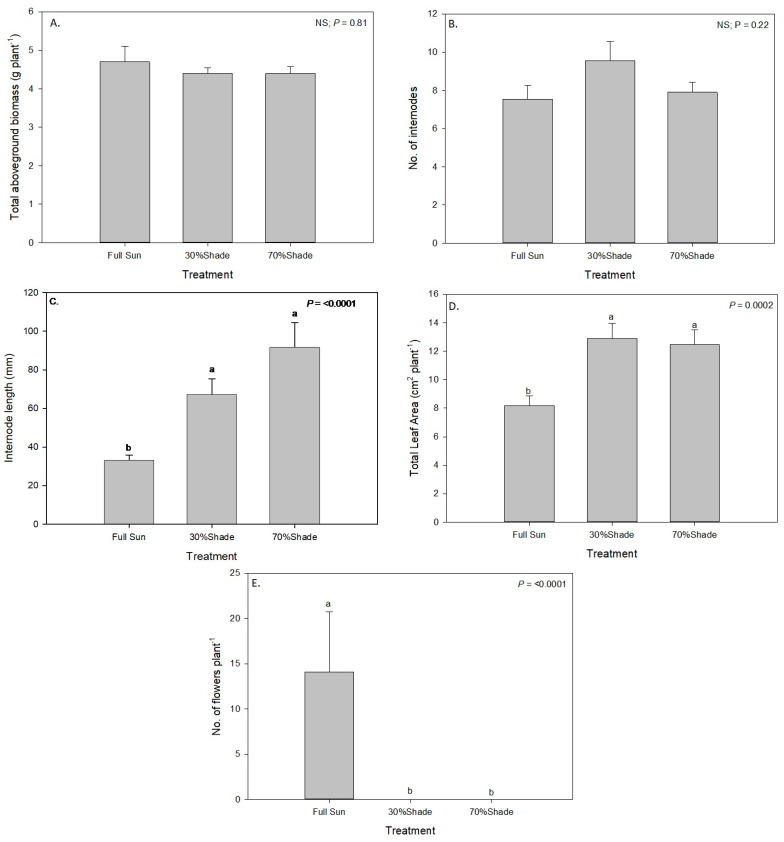
Effect of shade levels on (**A**) Aboveground biomass, (**B**) Number of nodes, (**C**) Internode length, (**D**) Total leaf area, and (**E**) number of flowers on alkaliweed at Stratford, CA, USA. Bars with the same lowercase letters are not significantly different at a 0.05 level of significance according to Fisher’s Least Significant Difference test.

The level of shade had an effect on the morphology, growth, and reproductive potential of alkaliweed plants. The photosynthetically active radiation (PAR) taken close to noon in the treatment plots during the course of the experiment, on average, ranged from 1700–2000, 1000–1300, and 500–700 µmol m^−2^ s^−1^ in the full sun, 30% shade, and 70% shade, respectively. Although the total aboveground biomass (Figure 7A) and the number of internodes on the stem (Figure 7B) were not affected by shade level, other morphological characters showed that this species was not a shade-tolerant plant and that plants exhibited efforts to adapt to shade. For example, the length of the internodes on the stem increased as the level of shade increased. The average length of the internodes in the plants growing in the full sun was approximately 35 mm whereas at 70% shade, it was approximately 90 mm (Figure 7C). Similarly, the total leaf area per plant was higher in the plants grown in the shade than in the full sun (Figure 7D). The total leaf area of the plants growing in the full sun was approximately 8 cm^2^ per plant whereas those in the shade had areas >12 cm^2^ per plant. Leaf thickness was not measured in the study, but it is suspected that leaves on the plants growing in the shade were comparatively thinner than on those growing in the full sun because thin leaves with less tissue per unit leaf area have been determined to be a characteristic of shade-intolerant plants growing in shaded environments as a result of etiolation [18]. Similar results were reported in field bindweed where internode length and total leaf area were lesser in plants grown in the full sun than those grown under shade [19]. However, field bindweed has long, viny stems and spreads more prostrately than alkaliweed.

Although the plants growing in the shade had similar aboveground biomasses as those in the full sun, none of the plants in either the 30% or the 70% shade treatments produced any flowers for the duration of this study (Figure 7E). Therefore, it appears that sexual reproduction can be severely inhibited in alkaliweed by shade and the use of shading strategies may be an effective management method to reduce sexual reproduction in this species.

### 3.5. Effect of Postemergence Herbicides on Alkaliweed

**Figure 8:**
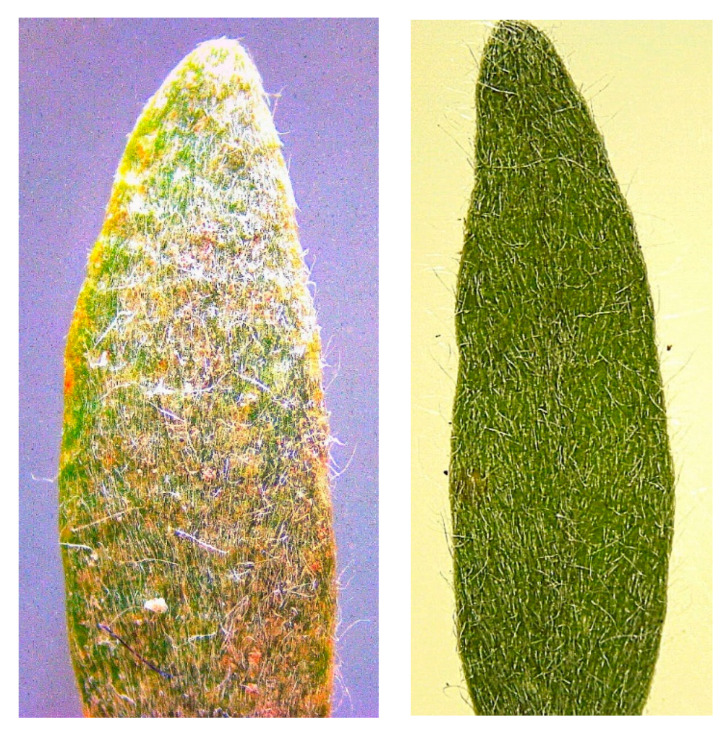
Microscopic images showing the presence of hairs on the adaxial side of the leaves of young alkaliweed plants.

Plant mortality differed between the herbicide treatments at each evaluation date (Table 2). Initially, saflufenacil (alone) and the tank mix of glyphosate + saflufenacil + glufosinate looked promising as more than 80% of the plants showed injury symptoms at 7 DAT and the plants appeared to be dying. However, by 28 DAT, the plants regrew, and the mortality was reduced to 50 and 53%, respectively, for the two treatments. This level of control would not be considered acceptable to a grower managing alkaliweed. None of the herbicide treatments provided acceptable control as the mortality rate was 20% or less in most cases. It was expected that the sequential application treatments would work better but this was not the case. Sequential applications of glyphosate and carfentrazone provided 43% control whereas that of glyphosate and paraquat provided only 15% control. Although appropriate adjuvants were applied in all the treatments, the postemergence herbicides failed to control the plants, which is perhaps due to the reduced contact, retention, and absorption of the herbicides because of the hairiness of the adaxial portion of the alkaliweed leaves as observed under a microscope (Figure 8). Even the leaves of small plants were observed to be hairy. In addition, it is not known if the regrowth is because of adequate levels of stored carbohydrates or the growth of new stems (aboveground or belowground) as discussed earlier. Therefore, alkaliweed control may not be feasible using postemergence herbicides alone. There are no published reports on alkaliweed control with herbicides; therefore, it is not possible to compare our results with other studies.

## 4. Conclusions

This study showed that the alkaliweed seeds could germinate in moderate drought and high salinity conditions under a range of soil pH. The species was not very shade-tolerant, and no sexual reproductive structures were observed in the plants growing in the 30 and 70% shade levels. None of the postemergence herbicides provided adequate control of the plants. The trichomes on the plant and leaf thickness make control of this plant with postemergence herbicides very difficult because it has been reported that these characteristics can affect the wetting and penetration of foliar-applied herbicides [20]. Therefore, further investigation on asexual reproduction ability and an integrated management plan need to be developed for alkaliweed control in the orchards of the Central Valley, CA, USA, and other semi-arid regions of the world where this species occurs as a problematic weed.

## Figures and Tables

**Table 1:** List of postemergence herbicide treatments.

No.	Treatment	Application Rate (ha^−1^)
1	Untreated control	-
2	Glyphosate (Roundup Powermax^®^) + AMS ^a^	1.38 kg ae ^b^
3	Glyphosate (Roundup Powermax^®^) + AMS	2.76 kg ae
4	Saflufenacil (Treevix^®^) + AMS ^a^ + MSO ^c^	70 g
5	Glyphosate (Roundup Powermax^®^) + AMS ^a^ followed by Paraquat (Gramoxone SL) + NIS ^c^ + AMS ^a^ applied 7 days later	1.38 kg ae + 3.65 L
6	Glyphosate (Roundup Powermax^®^) + AMS ^a^ followed by carfentrazone (Shark^®^) + MSO ^b^ + AMS ^a^ applied 7 days later	1.38 kg ae + 140 g
7	Glyphosate (Roundup Powermax^®^) + AMS ^a^ rimsulfuron (Matrix^®^) + NIS ^d^ followed by second application of the same treatments 14 days later	1.38 kg ae + 140 g 1.38 kg ae + 140 g
8	Glyphosate (Roundup Powermax^®^) + saflufenacil (Treevix^®^) + glufosinate (Rely 280^®^) + AMS ^a^	1.38 kg ae + 70 g + 4.09 L
9	US-15 n-Phurric acid	1:1 with H_2_O

^a^ Ammonium sulfate granular (AMS) @ 3.6 kg/378.5 l water. ^b^ acid equivalent (ae); ^c^ Methylated seed oil (MSO) @ 1% *v*/*v*. ^d^ Non-ionic surfactant (NIS) @ 1% *v/v*.

**Table 2:** Mortality of the alkaliweed plants to the various treatments at different days after treatment (DAT).

		Evaluation Date and % Plant Mortality ^a^			
No.	Treatment	7 DAT ^b^	14 DAT ^b^	21 DAT ^b^	28 DAT ^b^
1	Untreated control	0 d	0 d	0 f	0 e
2	Glyphosate (Roundup Powermax^®^)	10 c	20 c	10 ef	10 d
3	Glyphosate (Roundup Powermax^®^)	10 c	25 c	28 c	15 cd
4	Saflufenacil (Treevix^®^)	85 a	88 a	83 a	50 a
5	Glyphosate (Roundup Powermax^®^) + followed by Paraquat (Gramoxone SL) applied 7 days later	10 c	20 c	15 de	15 cd
6	Glyphosate (Roundup Powermax^®^) followed by carfentrazone (Shark^®^) applied 7 days later	13 c	48 b	60 b	43 b
7	Glyphosate (Roundup Powermax^®^) + rimsulfuron (Matrix^®^) + followed by second application of the same treatments 14 days later	10 c	18 c	18 cde	20 c
8	Glyphosate (Roundup Powermax^®^) + saflufenacil (Treevix^®^) + glufosinate (Rely 280^®^)	83 a	90 a	70 b	53 a
9	US-15 n-Phurric acid	40 b	43 b	23 cd	10 d
	*p*-value	<0.0001	<0.0001	<0.0001	<0.0001

**^a^** Evaluation scale: 0 = completely healthy without necrotic symptoms and 100 = dead with no green tissue remaining. ^b^ Means within a column with the same lowercase letters are not significantly different at a 0.05 level of significance according to Fisher’s Least Significant Difference test.

## Data Availability

The data generated in the experiment which supports the findings of this study are available on request from the corresponding author.
